# Identification of Adverse Drug Withdrawal Events after Older Adults Discontinue Statins

**DOI:** 10.1093/geroni/igaf122.3088

**Published:** 2025-12-31

**Authors:** Jonathan Norton, Glenn Goodrich, Jennifer Barrow, Valerie Paolino, Ariel Green, Orla Sheehan, Elizabeth Bayliss, Cynthia Boyd

**Affiliations:** Johns Hopkins University School of Medicine, Baltimore, Maryland, United States; Kaiser Permanente Colorado, Aurora, Colorado, United States; Kaiser Permanente Colorado, Aurora, Colorado, United States; Kaiser Permanente Colorado, Aurora, Colorado, United States; Johns Hopkins University School of Medicine, Baltimore, Maryland, United States; Royal College of Surgeons in Ireland, Dublin, Dublin, Ireland; Kaiser Permanente Colorado, Aurora, Colorado, United States; Johns Hopkins University School of Medicine, Baltimore, Maryland, United States

## Abstract

Assessing the safety of deprescribing interventions is difficult due to inadequate methods of accurately identifying adverse drug withdrawal events (ADWE) at scale. We explored methods to identify ADWEs after a medication discontinuation using electronic health record (EHR) data, and report on the relationship between statin discontinuations and potential ADWEs. The study population was older adults (65+), taking a statin, and had a confirmed statin discontinuation by chart review. Potential ADWEs were identified by ICD-10 and procedure codes (e.g. stroke, carotid artery disease, ischemic heart disease, coronary artery bypass graft, or percutaneous coronary intervention) within a 12-month time frame following the discontinuation. Two clinicians adjudicated each record to determine if the identified events were related to discontinuations and could constitute ADWEs. Outcome categories were: doubtful, possible, probable, or definite ADWE. Among 44 potential ADWEs, 48% (21/44) were determined to be doubtful (e.g. stroke during the 12 months following statin discontinuation was more likely related to atrial fibrillation than discontinuation), 48% (21/44) as possible (e.g. myocardial infarction (MI) occurred after discontinuation, but cancer may have also contributed to the MI), 4% (2/44) as probable (e.g. factors other than discontinuation couldn’t explain the ADWE), and 0% (0/44) as definite (e.g. same as probable, and confirmed improvement in medical condition after restarting of statin). These exploratory methods of identifying possible ADWEs with ICD-10 and procedure codes after a confirmed discontinuation, while still requiring record review, are new tools that could be enhanced and applied at scale in future deprescribing studies classes to assess safety.

